# Nanopipettes: probes for local sample analysis†
†Electronic supplementary information (ESI) available. See DOI: 10.1039/c5sc00668f



**DOI:** 10.1039/c5sc00668f

**Published:** 2015-04-13

**Authors:** Anumita Saha-Shah, Anna E. Weber, Jonathan A. Karty, Steven J. Ray, Gary M. Hieftje, Lane A. Baker

**Affiliations:** a Department of Chemistry , Indiana University , 800 E. Kirkwood Avenue , Bloomington , IN 47405 , USA . Email: lanbaker@indiana.edu

## Abstract

Nanopipettes (pipettes with diameters <1 μm) were explored as pressure-driven fluid manipulation tools for sampling nanoliter volumes of fluids. The fundamental behavior of fluids confined in the narrow channels of the nanopipette shank was studied to optimize sampling volume and probe geometry. This method was utilized to collect nanoliter volumes (<10 nL) of sample from single *Allium cepa* cells and live *Drosophila melanogaster* first instar larvae. Matrix assisted laser desorption/ionization-mass spectrometry (MALDI-MS) was utilized to characterize the collected sample. The use of nanopipettes for surface sampling of mouse brain tissue sections was also explored. Lipid analyses were performed on mouse brain tissues with spatial resolution of sampling as small as 50 μm. Nanopipettes were shown to be a versatile tool that will find further application in studies of sample heterogeneity and population analysis for a wide range of samples.

## Abstract 2

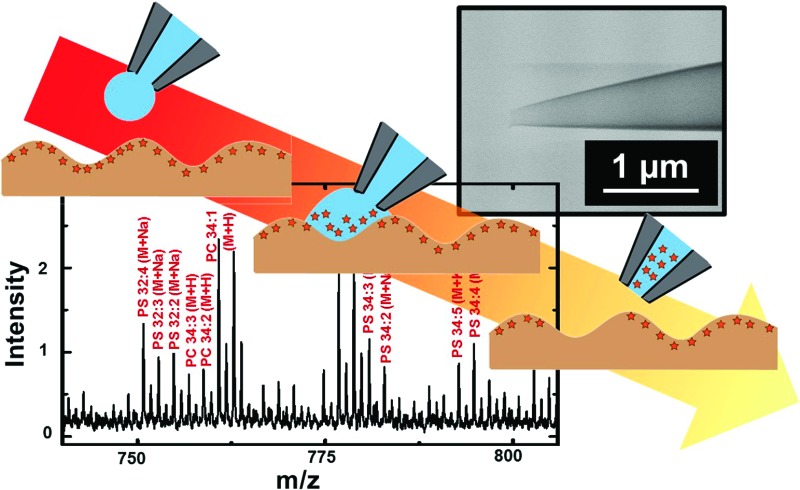
Nanopipettes are demonstrated as probes for local mass spectrometric analysis with potential for small-scale extraction of analytes from single cells, tissue and organisms.

## Introduction

Nanopipettes provide a simple – yet powerful – tool for manipulation of samples at small scales. A rich history that capitalizes on the ease of fabrication and unique nanoscale properties of nanopipettes exists.^1^ In particular, nanopipettes have been used in studies which include ion transfer,^2–6^ electrochemical imaging^7,8^ and delivery of controlled volumes.^9–11^ For instance, a seminal study by Mirkin and coworkers described an electrochemical syringe capable of voltage-controlled delivery of attoliter quantities of material into cells.^9^ This approach has recently been combined with scanning ion conductance microscopy to harvest and analyze genetic material of individual cells.^10^ In this report, we expand application of nanopipettes as sample collection devices and describe pressure-assisted manipulation of fluids with careful consideration of the impact of pipette geometry and dimension. We demonstrate analysis of nanopipette-sampled fluids *via* matrix assisted laser desorption ionization mass spectrometry (MALDI-MS) for biological samples at the level of single cells, tissues, and whole organisms.

The opportunity to couple nanopipette sampling with mass spectrometry is intriguing, as the impact of mass spectrometry coupled with soft ionization methods (*e.g.*, electrospray ionization (ESI), and MALDI), for chemical analysis cannot be overstated. In recent years, strategies that permit samples to be analyzed directly with minimal sample preparation have extended applications of MS. For instance, ionization methods such as desorption electrospray,^12,13^ paper spray,^14^ proximal probe thermal desorption^15^ and probe electrospray^16,17^ have demonstrated the possibilities of less complicated, chromatography-free analysis. Other approaches have demonstrated collection of sample material at very small scales, often making use of fluidic sampling interfaces such as liquid microjunction surface sampling probes,^18–21^ push–pull microfluidic devices,^22,23^ single-probe sampling^24–26^ and pipette sampling.^27–30^ In general, most (but not all^15,16^) of these techniques utilize probes that exceed a micron in size, and probes are typically on the order of ten microns or greater. Dimensions of sample collection (be it by laser, an electrospray source or a physical probe) remain a critical factor for applications such as mass spectrometric imaging. Shrinking probe dimensions promises to further expand applications of mass spectrometry as analysis moves toward routine single-cell and subcellular investigations.

Here we demonstrate that pipettes with tip dimensions <1 micron (so-called nanopipettes) can be used to reproducibly collect fluids with controlled pressure actuation. The geometry of the nanopipette is found to play a significant role in pressure-assisted sampling. Samples were collected *via* nanopipettes from single *Allium cepa* cells, first instar larve of *Drosophila melanogaster* and from sectioned brain tissue of a mouse. Fluids collected were subsequently subjected to MALDI-MS analysis, and results agree well with previously reported MS analysis^31–35^ of similar samples. Advantages of nanopipettes in this role are underscored particularly in the case of brain tissue analysis, where both spatial resolution of the method is found to be finer than other approaches (such as touch spray sampling^17^) and simple liquid extraction (*i.e.* the Folch method^36^) can be incorporated for analysis. We propose that the general availability, simplicity and applicability of nanopipettes represent an attractive alternative for micro-nano sampling for future mass spectrometry applications.

## Experimental

### Chemicals and materials

Aqueous solutions for mass spectrometry experiments were prepared with deionized water (18 MΩ cm) obtained from a Milli-Q water-purification system (Millipore Corporation, Danvers, MA); methanol was obtained from Mallinckrodt Chemicals (St. Louis, MO). MALDI matrix 2,5-dihydroxybenzoic acid (DHB), dextran and internal standards for high mass accuracy such as PEG 400 and PEG 1000 were purchased from Sigma-Aldrich (St. Louis, MO). All chemicals were used as received. Quartz capillaries for fabrication of nanopipettes were purchased from Sutter Instrument (Novato, CA). Procedures for nanopipette fabrication and characterization are detailed in the ESI.†

### Pressure-assisted nanopipette sampling

A manual micromanipulator (World Precision Instruments, Sarasota, Florida) was used to position nanopipettes for the aspiration experiments. Pipettes were connected to a vacuum-tight ‘T’ connector (Cole Parmer, Vernon Hills, IL) through polyethylene tubing; the second and third ports of the ‘T’ connector were attached to a pressure monitor (Vaccon vdx (VDXN-QD-6) electronic vacuum switch, Medway, MA) and a gas-tight syringe, respectively. The gas tight syringe plunger was manually pulled to apply negative pressure for aspiration and pushed to apply a positive pressure for dispensing. For sampling experiments a negative pressure was applied for one minute, after which time the pipette was imaged with an upright optical microscope to assess solution ingress. Pipettes remained intact after aspiration experiments, with tip diameters verified by scanning transmission electron microscopy. Aspirated volume (*V*) was calculated based on geometry of the nanopipette from eqn (1), adopted from Mirkin and co-workers.^9^
1
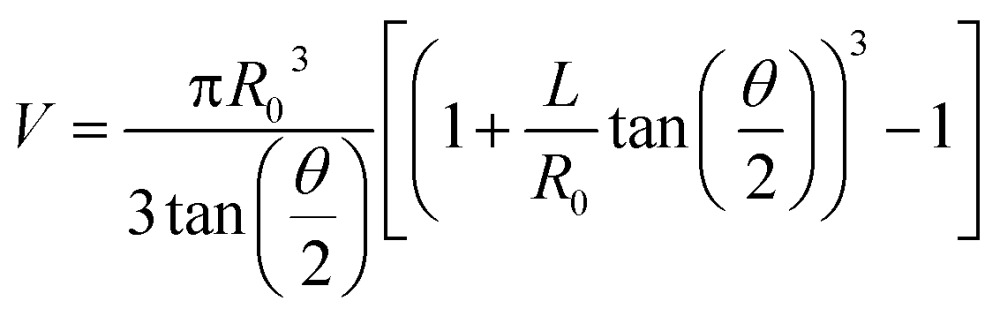
Here, *R*_0_ is the tip diameter, *θ* is the pipette cone angle and *L* is the height of solution ingress.

### Mass spectrometric analysis

A Bruker Autoflex III MALDI-TOF Mass Spectrometer equipped with a frequency-tripled Nd:YAG laser (355 nm) was used in reflectron positive-ion mode. Analytes were transferred to a MALDI plate by application of positive pressure to the nanopipette. Matrix solution was dropcast on the MALDI target plate (AnchorChip MTP 400/384, Bruker Daltonics) over the dried analyte spot as per the manufacturer's instructions. A 20 mg mL^–1^ DHB in 50% methanol–water was used for *A. cepa* cytoplasm analysis and mouse brain tissue section analysis was performed with 70 mg mL^–1^ DHB in 50% methanol–water. A 8 mg mL^–1^ dithranol matrix in 2 : 1 chloroform/methanol^32^ was used for *D. melanogaster* hemolymph analysis.

### *Allium cepa* sample preparation

Fresh red onions (*A. cepa*) were obtained from a local market. The epidermis was peeled with tweezers and mounted on a clean glass slide. Cells were located and punctured with a nanopipette under an optical microscope. The cell cytoplasm was aspirated by application of a negative pressure for *ca.* 30 s. Optical microscopy was utilized to determine the volume of cytoplasm collected.

### *Drosophila melanogaster* hemolymph collection

*D. melanogaster* w1118 type larvae were a generous gift from Prof. Justin P. Kumar (Indiana University). Larvae were reared on standard cornmeal-agar medium. First instar larvae were identified on the basis of mouth hook and spiracle morphology. Prior to hemolymph collection, larvae were washed with purified Milli-Q water to remove adherent food, and then blotted dry. Live larvae were affixed to a glass slide with double-sided tape and mounted on a microscope stage. For hemolymph collection, the cuticle of each larva was punctured with a nanopipette and negative pressure was applied.

### Preparation of mouse brain tissue sections for lipid analysis

All experiments on the mouse brain were performed in accordance with protocols approved by the Bloomington Institutional Animal Care and Use Committee at Indiana University. Harvested tissue was immediately frozen in dry ice and stored at –80 °C. Sectioning was carried out by mounting the tissue section onto the cryostat chuck with OCT (optimum cutting temperature compound). The frozen mouse brain was sectioned at –20 °C with a cryostat microtome (AO Reichert Scientific Instruments, Buffalo, NY, model: 975C). Serial tissue sections of 15 μm thickness were collected on polydimethylsiloxane (PDMS) coated glass slides and stored at –80 °C until sampling experiments. About 30 min before sampling experiments, tissue sections were removed from –80 °C, warmed to room temperature in a desiccator, and were then mounted in an optical microscope. A nanopipette of ∼750 nm I.D. (inner diameter) filled with 2 : 1 chloroform/methanol solution (Folch method) was approached to the white matter of the brain tissue section and positive pressure was applied to dispense solution. Analyte molecules were thereby extracted into the organic solvent (dispensed from the pipette) from a small (50 × 50 μm) portion of the tissue section. Subsequently, a negative pressure was applied to aspirate the organic solvent and extracted materials into the pipette. This procedure (of alternate aspiration and collection) was repeated 3–4 times to extract biomolecules from a small section of the tissue. Extract was deposited on the MALDI plate by application of positive pressure, followed by application of DHB matrix and MALDI-MS analysis. A serial section of the brain tissue was fixed and stained with H&E (hematoxylin and eosin) stain to identify the location sampled from the previous section.

## Results and discussion

### Sample aspiration

**Fig. 1:**
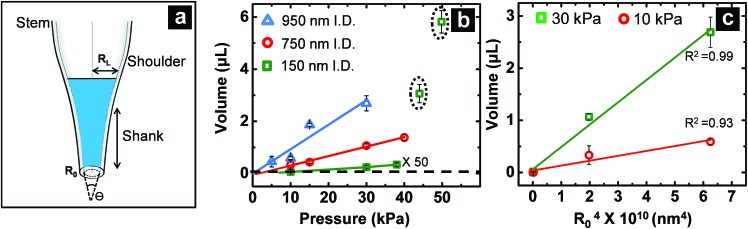
(a) Illustration of a nanopipette that indicates geometric parameters such as tip radius (*R*_0_), shank length (*l*), shoulder, radius inside the pipette after volume ingress (shaded in blue) (*R*_*L*_) and cone angle (*θ*), (b) volume of sample aspirated as a function of pressure for tip diameter ∼950 nm (I.D.) (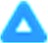), ∼750 nm (I.D.) (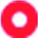), and ∼150 nm (I.D.) (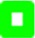). (c) Plot of volume aspirated against (*R*_0_)^4^ at a constant pressure of 30 kPa (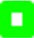) and 10 kPa (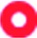). For all plots, *n* = 3, where *n* corresponds to three independent measurements with different nanopipettes of similar dimensions.

For pressure-assisted sampling experiments, aspiration time was held constant at one minute, and pressure was varied to control volume of aspirated sample. Optical micrographs (Fig. S1†) and volume calculations (Table S1†) demonstrate that aspirated sample volumes were reproducible under consistent experimental conditions. The small variation in aspirated volume (∼12% RSD, Table S1†) can be attributed to slight differences in pipette geometry (geometric parameters of a pipette are illustrated in Fig. 1a). For most pipettes, the sampled volume was reproducible at nanoliter- to microliter-scales and increased with applied pressure as predicted by the Hagen–Poiseuille equation.^37^ However, non-linearity of sampled volume was clearly evident in pipettes with ∼150 nm I.D. (Fig. 1b). Volumetric flow increased in a relatively monotonic fashion until 38 kPa, after which a sudden increase in volumetric flow rate was observed. To evaluate the origin of such anomalous flow behavior, a modified Hagen–Poiseuille equation was applied and found to be an appropriate model to describe fluid flow through the nanopipette shank. Traditionally, the Hagen–Poiseuille equation is used to correlate volumetric flow rate (*W*) and pressure drop (Δ*P*) for a Newtonian fluid in a cylindrical tube of constant radius (*R*). The Hagen–Poiseuille equation, modified to accommodate flow through a tapered tube, such as the shank of a nanopipette, is given by eqn (2):^37,38^
2
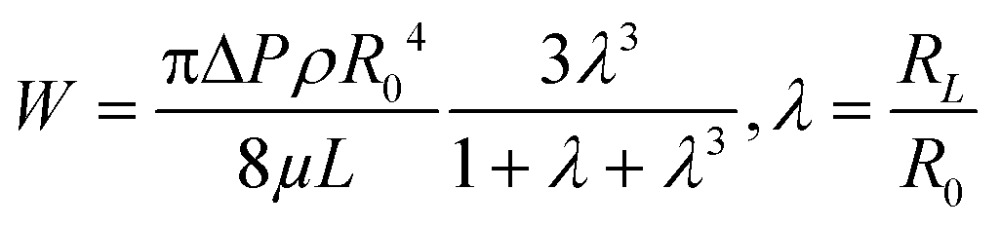
where, *ρ* is the fluid density, *μ* is fluid viscosity, *L* is the height of volume ingress, and *R*_0_ and *R*_*L*_ refer to the inner radii of the tapered tube at the tip and at height ‘*L*’, respectively. Reynolds numbers (Re) were calculated as described by van Dongen^39^ and found to be <100 for the flow regimes sampled in these experiments. Thus laminar flow, an essential condition for Hagen–Poiseuille flow, was confirmed. Nanopipettes of various radii were employed to aspirate samples at a constant pressure difference. The volume aspirated for each sample was plotted as a function of (*R*_0_)^4^ (Fig. 1c) for a constant-pressure difference of 30 kPa (green squares) and 10 kPa (red circles). A linear trend was observed for both plots, in agreement with eqn (2). Of note, the coefficients of determination (*R* ^2^) for the linear plots in Fig. 1c were obtained from only four data points; the initial two points lie close to the origin of the plot and can be distinguished in Fig. S2† (aspirated volume *vs.* nanopipette radius). In accordance with eqn (2), the taper function, *λ*, is a function of *R*_0_, which usually leads to non-linearity in volume *versus* (*R*_0_)^4^. We observed linear behavior for volume *versus* (*R*_0_)^4^ and postulate that at low pressure (<38 kPa) the 
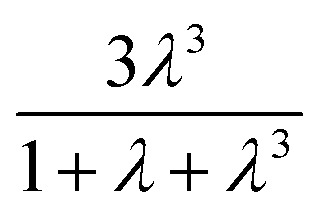
 term in eqn (2) does not contribute significantly to the volume sampled when fluid is confined to the narrow shank of the pipette where *θ*, the cone angle, is very small and for practical purposes the nanopipette can be considered a cylindrical tube. As a result, the volume *versus* (*R*_0_)^4^ plot does not deviate from linearity (Fig. 1c).

### Impact of nanopipette geometry on fluid manipulation

**Fig. 2:**
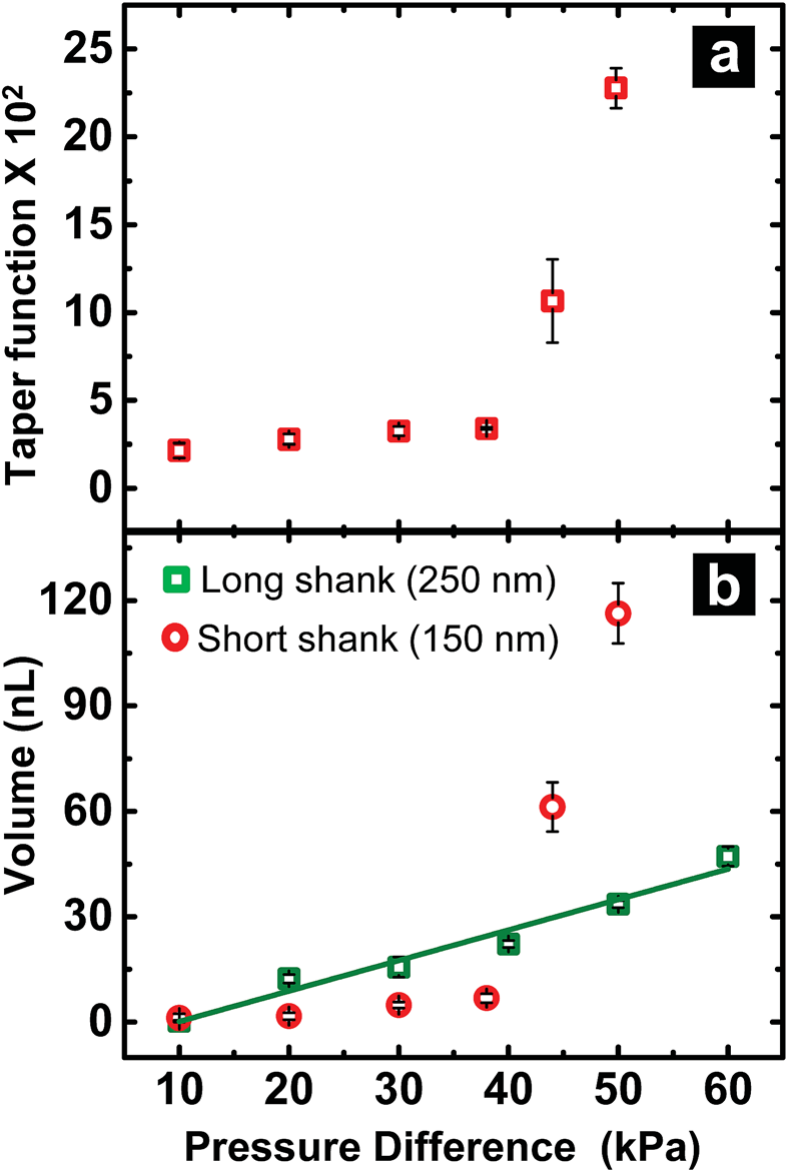
(a) Variation in taper function with pressure for ∼150 nm short shank pipettes. (b) Volume of sample aspirated as a function of pressure difference for long (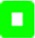) and short (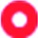) shank pipettes. For all plots, *n* = 3.

When fluid ingress reaches the shoulder region of the nanopipette, the volume collected for ∼150 nm I.D. pipettes deviate, as highlighted (with black dotted line) in Fig. 1b at Δ*P* > 38 kPa, (shown in Fig. S3a†). This observation is supported further by similarity in relationship between pressure and both calculated taper function (from experimentally determined *R*_0_ and *R*_*L*_ post-sampling) (Fig. 2a) and experimentally measured volume sampled (Fig. 2b). To study the effect of pipette geometry on mass flow, another set of pipettes with long shanks (long taper with small cone angle) were fabricated (Fig. S3d–S3f†). The I.D. of these pipettes, prepared by focused ion-beam milling (FIB), were ∼250 nm (please note, FIB was only used to adjust the size of the tip opening as long shank pipettes with >100 nm I.D. could not be obtained by the laser puller. A 60 nm I.D. tip was fabricated and FIB milled to obtain 250 nm I.D. pipettes). Although an ideal comparison of long-shank *versus* short-shank pipettes would use pipettes of identical diameter, fabrication of long-shank pipettes of 150 nm I.D. proved experimentally prohibitive.

Fig. 2b shows variation in sampled volume with pressure difference for the two sets of nanopipettes (time of aspiration was kept constant). Long-shank pipettes exhibited a linear relationship over the range of pressures studied, unlike short-shank pipettes. Close inspection of optical micrographs of long-shank nanopipettes (Fig. S3d–S3f†) revealed that samples did not enter the shoulder region even at a pressure difference of 50 kPa. This behavior is in contrast to that of short-shank nanopipettes (Fig. S3a–S3c†); as a result, long-shank pipettes, which have smaller *R*_*L*_ values, exhibited a linear relationship between sampled volume and pressure. With this in mind, the linear nature of response for long-shank pipettes proved beneficial for further small volume sampling experiments, as precise control of sampling could be obtained over a wider range of volumes.

### Correlation of hydrodynamic resistance to nanopipette tip size

**Fig. 3:**
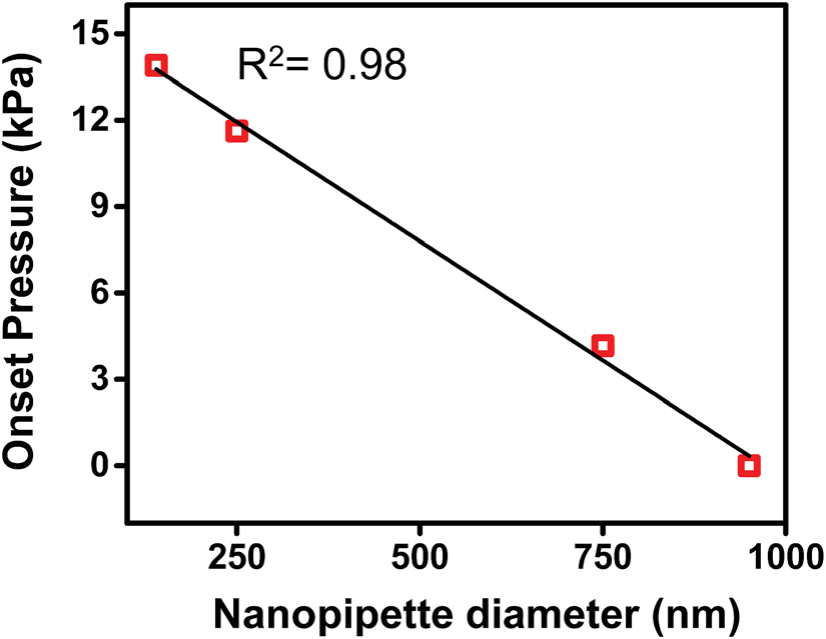
Plot of onset pressure (obtained from *X*-axis intercept of Fig. S4†) *versus* tip diameter.

In Fig. 2b, both traces were observed to have an *x*-axis intercept. To rationalize the *x*-axis intercept, the initial sample ingress due to capillary action was determined (see Table S2†) and subtracted from the final volume to compare sample ingress due solely to pressure-driven aspiration for pipettes of tip I.D. ∼150 nm, ∼250 nm, ∼750 nm, and ∼950 nm. *X*-axis intercepts were observed for three of the traces (where nanopipette I.D. was ∼150 nm, ∼250 nm, and ∼750 nm) which represents the minimum pressure necessary to achieve any pressure-driven aspiration. The minimum onset pressure was found to correlate inversely to tip diameter (Fig. 3) for the tip dimensions studied here, which indicates the resistance for fluid flow into these pipettes depends strongly on tip diameter. This correlation is in agreement with previous studies of pressure-driven micropipette injections where the sum of all resistances encountered by the fluid at the micropipette tip was referred to as the hydrodynamic resistance.^39^ The linear trend between nanopipette radius and onset pressure (for the pipettes over the dimensions studied here) suggests that an increase in size of the nanopipette tip lowers the resistance and hence lowers the minimum onset pressure. This result is particularly significant and can in future be used to the advantage of the user to manipulate lower volumes by employing even smaller nanopipette tips. Please note that the flow rate for small tips could be significantly low as flow rate is inversely proportional to *R*_0_^4^ and onset pressures could potentially reach very high values.

### Analysis of cytoplasm from single *Allium cepa* cell

**Fig. 4:**
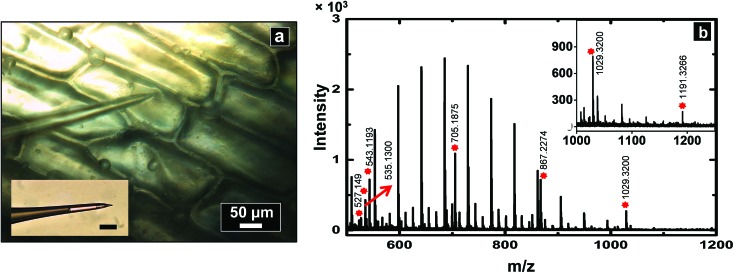
(a) Optical micrograph which shows a nanopipette used to sample cytoplasm from an *A. cepa* epidermal cell. Inset shows an optical micrograph of the pipette post-sampling (scale bar: 50 μm). Sampled volume was calculated to be ∼8 nL, and was analyzed by positive ion MALDI-TOF mass spectrometry to obtain the mass spectrum as shown in (b). The peaks labelled by a red asterisk are metabolites obtained from cells, the unlabelled high intensity peaks correspond to Triton X-100 added for internal calibration.

To demonstrate application of this technique for single cell analysis, cytoplasm of *A. cepa* cells were sampled with a nanopipette of 600 nm I.D. and analyzed by MALDI-MS (see spectrum Fig. 4b) for hydrophilic metabolites such as oligosaccharides. Peaks were tentatively assigned based on mass matches (mass accuracy <25 ppm) to metabolites previously identified in *A. cepa*.^31,33,40^ Polyethylene glycol (PEG)-400 was added to the sample spot to calibrate the spectrum from *m*/*z* = 409.1840 to 595.2889, Triton X-100 for *m*/*z* 537.3398 to 1021.6282, and PEG 1000 for *m*/*z* 849.4462 to 1509.8394. A list of metabolites identified is provided in the ESI (Table S3†). Low molecular weight fructans are the major storage carbohydrates and found in abundance in red onions. A series of peaks were observed with mass difference of 162 Da (mass of hexose units), suggestive of the presence of hexose-oligosaccharides. On the basis of accurate mass measurement, prominent peaks were assigned to hexose-oligosaccharides with degrees of polymerization 3 to 7 (MALDI-MS analysis cannot distinguish between isomers such as fructans and glucans and hence the peaks were assigned generally as oligosaccharides). The identity of the oligosaccharides was further supported by spiking the samples obtained from *A. cepa* with standard dextran oligosaccharides of ∼*M*_r_ 1500 (see spectra in section S6 of ESI†). Since onion bulbs contain high levels of potassium, the oligosaccharide peaks are observed as potassium adducts, which is consistent with previous reports on carbohydrate analysis of *A. cepa*.^33^

### Analysis of *Drosophila melanogaster* hemolymph

**Fig. 5:**
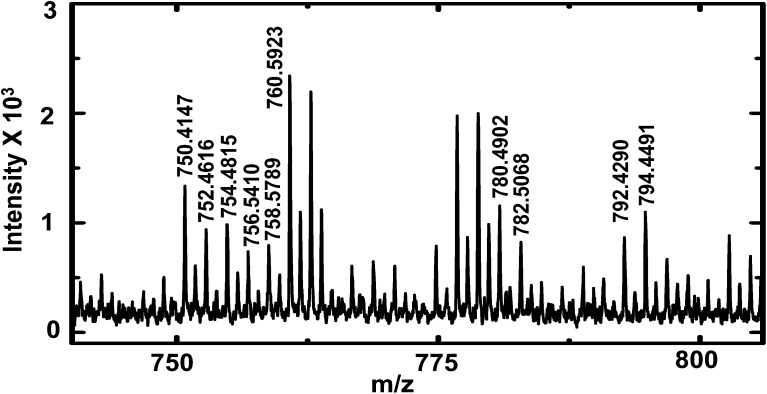
MALDI-TOF mass spectra of *D. melanogaster* first instar larva hemolymph showing lipid peaks (labelled). A list of lipids observed is provided in ESI† (Table S4).

To demonstrate the versatility of this sampling technique for the analysis of complex biological samples, hemolymph of first instar *D. melanogaster* larvae were sampled and analyzed by MALDI-MS for lipid composition. *D. melanogaster* is commonly used to study the function of bioactive lipids, but limited information on molecular identity of these lipids hampers structure activity correlation.^41^ The study of lipid composition is particularly important because of the relationship between lipid composition and disease states.^42^ Increased knowledge of lipid composition heterogeneity among organisms of the same phenotype through analysis of individual larva will provide crucial information about metabolic processes.^41^ Earlier reports have described analysis of the chemical composition of individual third instar larva^43,44^ and adult flies.^32^ To study hemolymph from earlier stages (compared to third instar larvae) with a lower hemolymph volume (∼70–100 nL, hemolymph volume of first instar larva estimated from size), a more appropriate tool for extraction of hemolymph is required. The method described here is appropriate for sampling and analysis of small volumes from first instar larvae. A small puncture was made in the cuticle of the larva with a 600 nm I.D. nanopipette to aspirate hemolymph. Care was taken not to puncture and aspirate hemolymph from a region close to the gut (observed under optical microscope as a thin black tube) because application of negative pressure was found to result in clogging of the pipette by the gut structure. After sample aspiration was complete, the pipette tip was retracted and disconnected from the pressure-sampling apparatus; an image of the sampled volume inside the nanopipette tip was then acquired and used to calculate the sampled volume. Sampled hemolymph was then deposited on the MALDI target plate, followed by application of matrix and MALDI-MS analysis (see Fig. 5).

Peaks were tentatively assigned based on a search in the LIPID MAPS Lipidomics Gateway database^45^ with ∼25 ppm mass accuracy. Internal calibration (for higher mass accuracy) was performed with PEG 600 and a list of lipids identified is provided in the ESI† (Table S4). The lipids identified matched literature reports of lipid composition of adult *D. melanogaster*.^32,34^ Phosphatidylcholines (PC) of overall carbon chain length 28 to 38 are found in abundance in *D. melanogaster.* ^32^ In this study, we observe the presence of PCs with carbon chain length of 34 and with various degrees of unsaturation. Some peaks were tentatively assigned to phosphatidylserines of carbon chain length of 32 and 34, and were observed as both sodium and potassium adducts. Of note, the list of lipids identified in this study is not exhaustive, other peaks likely due to lipids were observed in the mass spectra, but their mass errors were larger than 25 ppm and hence were not included in Table S4.† The purpose of this preliminary study was to demonstrate the effectiveness of sampling first instar *D. melanogaster* larva hemolymph with nanopipettes and to demonstrate the utility of our sampling technique for analysis of complex biological mixtures.

### Lipid analysis in mouse brain

**Fig. 6:**
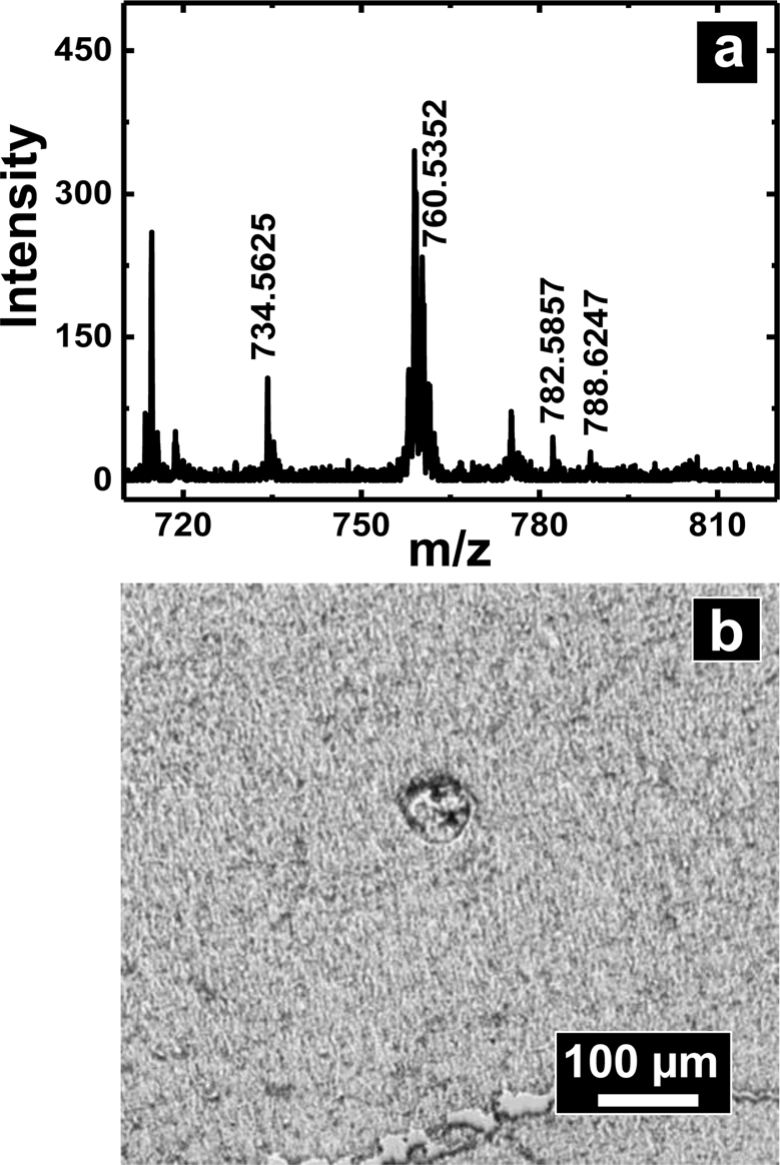
(a) Positive ion MALDI-MS of extracted lipids from a ∼50 × 50 μm spot of mouse brain tissue section. (b) Optical micrograph of the brain tissue section from which lipids were extracted. Indicated with the dashed white circle is the damage created by solvent extraction.

A slightly different approach was used to sample from flat tissue sections with nanopipettes. Tissue section sampling was performed with a secondary solvent to selectively desorb analytes of interest. Lipid analysis from mouse brain was the primary focus and to accomplish that, the Folch method was adopted. Nanopipettes of ∼750 nm I.D. were filled with the secondary solvent (chloroform/methanol mixture) and a small drop of 0.5–1.5 nL solution was created at the tip of the pipette, the drop was then deposited and subsequently aspirated back into the pipette. This process was repeated 3–4 times to ensure efficient extraction of desired analyte into the secondary solvent. The drop size formed at the tip of the nanopipette is correlated to the diameter of the analyzed spot. Control of pressure and tip size was used to reproducibly obtain spots of diameter 50–1000 μm. The spot size of sampling was determined from the mark as shown in Fig. 6b created by the solvents due to contact with the tissue section.

Shown in Fig. 6a is a mass spectrum of lipids extracted from a 50 × 50 μm spot of white matter in mouse brain as identified by a H&E stained serial section. Phosphatidylcholine peaks were observed primarily in the positive ion mass spectrum. Mass spectral peaks were assigned based on accurate mass and previous reports^35^ of lipid analysis from mouse brain. A list of lipids observed is provided in Table S5 of ESI.† The lipids observed include PC 32:0, PC 34:1, PC 36:1, and PC 36:4 which are found in abundance in cerebral cortex. The work on tissue surface sampling presented here is a prelude to the utility of nanopipettes for localized sampling from tissue sections with spot sizes of <100 μm as compared to present state-of-the-art surface sampling techniques such as LESA^19^ (liquid extraction surface analysis) and PLESA^20^ (pressurized liquid extraction surface analysis), which have sampling spot sizes >400 μm. More detailed studies to build spatial maps of lipids and other metabolites from tissue sections are presently underway.

Peaks other than those of oligosaccharides, such as *m*/*z* 527.1424 and 535.1300 from *A. cepa* and lipids from *D. melanogaster* and mouse brain sections, are tentative assignments made with the aid of previous detailed analysis by mass spectrometry^31–35^ and should be considered as a proof-of-concept study to demonstrate utility of the technique rather than definitive assignments. MS^n^ analysis may be necessary for definitive assignments.

## Conclusion

Nanopipettes were utilized as a local probe for sample collection and manipulation. The sampling method is relatively straightforward and collected samples were not diluted, contaminated, or altered by the presence of electrolytes or application of high voltage, as can be the case with other sampling techniques.^9,27^ The fundamental behavior of fluid confined in a narrow, tapered channel (shank) of a nanopipette was studied, which revealed that flow rate is dependent on nanopipette geometry and tip diameter. Results described here can be utilized to tune the probe size and geometry of nanopipettes for manipulation of desired volumes. Complex biological samples such as single *A. cepa* cells, *D. melanogaster* hemolymph and mouse brain tissue sections were analyzed with matrix-assisted laser desorption/ionization mass spectrometry for identification of oligosaccharides and lipids. Thus, this technique is appropriate for analysis of a variety of hydrophobic and hydrophilic analytes from different matrices. In future studies the ultimate limits of detection and minimum volumes that can be reasonably sampled will be studied with smaller tip sizes (with higher hydrodynamic resistance) and application of pressure for very short durations. Moreover, use of positive pressure can completely thwart ingress due to capillary action and afford more controlled sample collection from specific locations in a sample. Better control over small volume manipulation can enable sampling from smaller spot sizes in tissue sections and also provides a route to sequential and selective extraction of desired analyte from highly localized regions. In this initial report, we have demonstrated spatial sampling from a tissue section that is relatively high resolution for MS sampling. In the future, full use of the nanometer scale dimensions and positioning of nanopipettes will be explored through feedback-based pipette positioning, which is expected to improve the spatial resolution further. As shown here, nanopipettes provide an appealing method to sample small volumes and hold significant potential for study of population heterogeneity especially when coupled with scanning probe microscopy techniques. Also, potential for a wide range of approaches to sample pre-treatment and selective enrichment of analytes can be explored.
