# Supplementary material for: Nanopipettes: probes for local sample analysis
Source: Chem Sci. 2015 Apr 13;6(6):3334–41. doi: 10.1039/c5sc00668f (PMC5490420; doi:10.1039/c5sc00668f)
Supplement: Supplementary file 1 [file SC-006-C5SC00668F-s001.pdf]

Electronic Supplementary Material (ESI) for Chemical Science.
This journal is © The Royal Society of Chemistry 2015

Supporting Information

S1 Pipette fabrication and characterization

Quartz capillaries with 0.7-mm inner diameter (I.D.) and 1.0-mm outer diameter (O.D.) were used to fabricate ~150 nm and ~250 nm pipettes with a CO_2 laser-based pipette puller (P-2000, Sutter Instrument, Novato, CA). Pipettes with I.D. ~750 nm and ~950 nm were fabricated from quartz capillaries with 0.3-mm I.D. and 1.0-mm O.D. Prior to nanopipette fabrication, capillaries were treated with piranha solution (H_2O_2:H_2SO_4 = 1:3) to remove organic contaminants.

Nanopipette tip diameter and cone angle were characterized with scanning electron microscopy (SEM) and scanning transmission electron microscopy (STEM) images obtained with a Quanta-FEG 600F scanning electron microscope (FEI, Hillsboro, OR). Image processing and analysis were performed with ImageJ (National Institutes of Health, Bethesda, MD).

S2 Focused Ion Beam (FIB) milling of nanopipettes

A Zeiss Auriga® Modular Cross Beam work station (Oberkochen, Germany) was used to mill bare quartz nanopipettes to obtain long shank (taper) nanopipettes with a 250 nm I.D. The ‘Mill for depth’ function was selected in the SmartSEM® V05.05 XB operating software. A 5 µm depth and a 30 kV, 500 pA beam current was used.

S3 Sample volume reproducibility

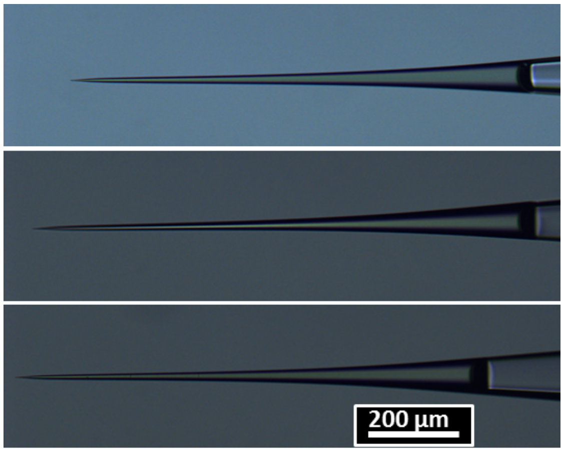

Figure S1 Optical micrographs of three nanopipettes (same size and geometry) with purified water aspirated at a pressure difference of 44 kPa (aspiration time = 1 minute).

Table S1 List of pipette parameters and volume of sample aspirated, of the pipettes shown in Figure S1, at identical conditions of pressure, time and solution viscosity.

Pipette #	Radius (nm)	Height of ingress (µm)	Cone angle(°)	Volume (nL)	Average volume (nL)	Standard Deviation (nL)	RSD (%)
1	72	1007	12	53	61	7	12
2	70	1041	14	66			
3	75	1014	13	63			

S4 Behavior of fluids inside nanopipette shank and tip

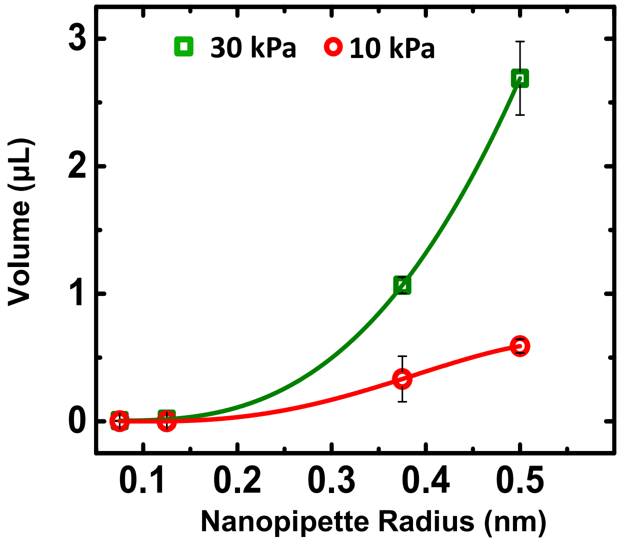

Figure S2 Volume aspirated versus nanopipette radius at a constant pressure difference of 30 kPa (▣) and 10 kPa (⊙). Arbitrary lines were drawn to connect the data points.

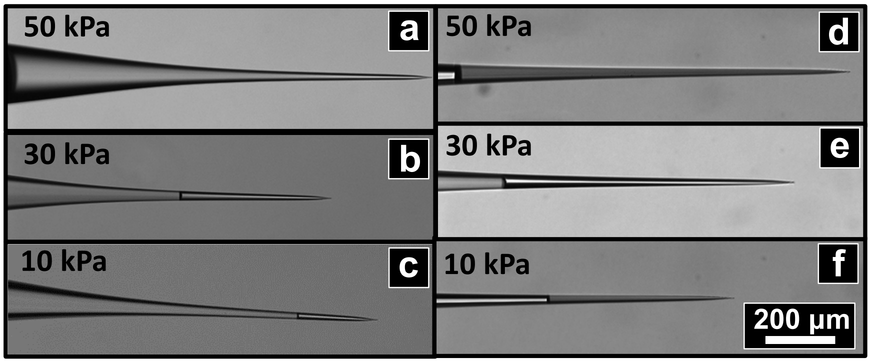

Figure S3 Optical micrographs of ~150 nm (I.D.) short shank nanopipettes (a-c) and ~250 nm (I.D.) long shank nanopipettes (d-f) when subjected to pressure-assisted sampling of purified water at various pressures.

Table S2 List of nanopipette tip diameter and water ingress (inside nanopipette) due to capillary action.

Nanopipette tip diameter (nm)	Ingress due to capillary action (nL)
150	0.0064
250	0.01
750	0.03
950	1.02

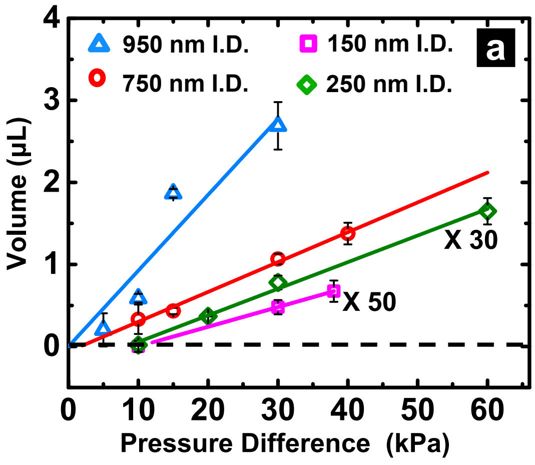

Figure S4 Volume of sample aspirated as a function of pressure difference plot for tip diameter ~950 nm (I.D.) (△), ~750 nm (I.D.) (○), ~250 nm (I.D.) (◇), ~150 nm (I.D.) (□). Ingress of sample due to capillary action was subtracted from the total volume for this plot to compare ingress due to pressure driven aspiration only.

S5 Mass spectrometric analysis of standard sample aspirated with nanopipettes

Prior to application of the nanopipette sampling technique for mass spectrometric analysis of real biological sample such as *Allium cepa* and *Drosophila melanogaster*, standard mass spectrometry analyte solution was sampled and corresponding mass spectrum was acquired (**Figure S5**) to validate the pipette sampling method. For this analysis, sample deposition was performed by immersing the nanopipette in a drop of water, followed by application of a positive pressure. In this experiment, the sample delivery on to MALDI plate was performed by immersing the tip into a drop of water so as to maintain the integrity of the tip for subsequent STEM imaging. After sample deposition, STEM images of the tips were acquired and the integrity of the pipettes was verified. Determination of tip integrity is an important step to ensure the nanopipette tips do not break during sampling and the samples collected (and hence the mass spectrum) were obtained from a localized region of a sample.

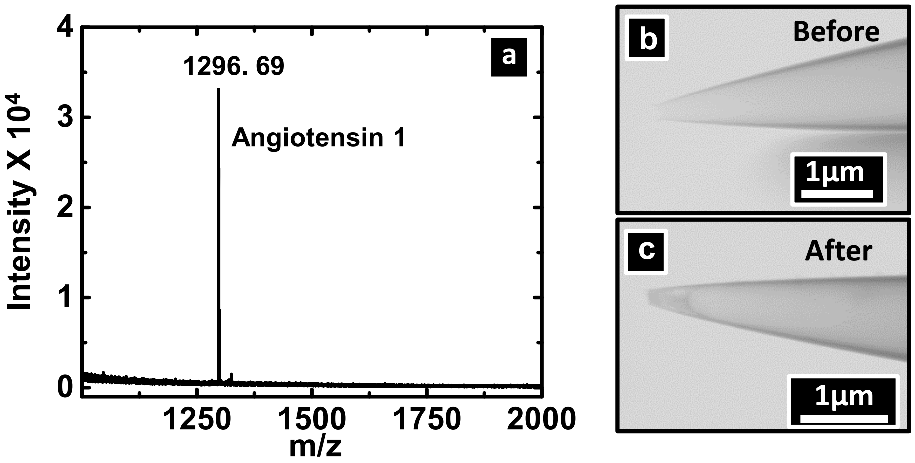

Figure S5 MALDI-MS spectra of 20 µM solution of angiotensin I aspirated into a 112 nm I.D. nanopipette. Volume of analyte aspirated was 32 nL, or 644 fmol, of angiotensin I. (Matrix: α-cyano-4-hydroxycinnamic acid). Scanning transmission electron micrograph

of the nanopipette before sampling (b) and after sampling (c). The I.D. before and after sampling was ~112 nm.

Table S3 Tentative Peak assignments in mass spectrum of a single *Allium cepa* epidermal cell.

**Assignment**	**Exact mass**	**Observed mass**	**Δppm**
Glutamine+Sucrose+K	527.1490	527.1424	12.5
Cyanidin malonyl glucoside	535.1087	535.1300	39.87
Trisaccharide+K	543.1328	543.1193	24.8
Tetrasaccharide+K	705.1875	705.1815	2.6
Pentasaccharide+K	867.2384	867.2274	12.6
Hexasaccharide+K	1029.2913	1029.3200	27.8
Heptasaccharide+K	1191.3440	1191.3266	14.6

S6 Spiking study to further support the identity of oligosaccharides

For the spiking studies, a standard solution of dextran was prepared in water and was analyzed by MALDI-MS. Another spot of *Allium cepa* cytoplasm was prepared and analyzed for oligosaccharides independently by MALDI-MS after application of DHB matrix. Then the spot was spiked with above mentioned dextran stock by application of a microliter of the standard onto the dried cytoplasm/matrix spot. The spot was again analyzed for oligosaccharides under identical conditions as the previous spot. Shown below are some of the representative mass spectra of a sample before (in green) and after (in black) spiking with dextran standard. The spectra were zoomed-in to confirm the presence of only one peak at m/z corresponding to the oligosaccharides. Ofnote, for the black trace, internal calibration was not performed. The green trace was internally calibrated as per the procedure detailed in the paper.

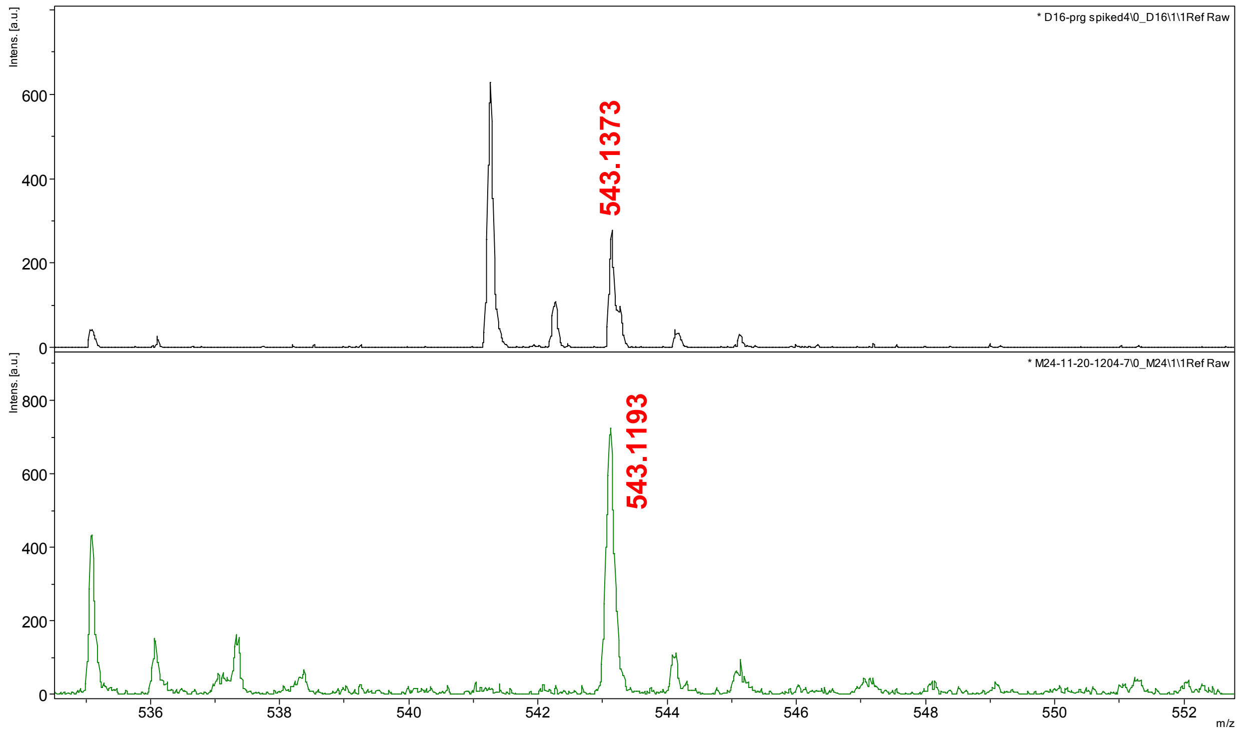

Figure S6 MALDI-MS spectra of trisaccharide+K in *Allium cepa* cytoplasm before (green) and after (black) spiking with dextran standard.

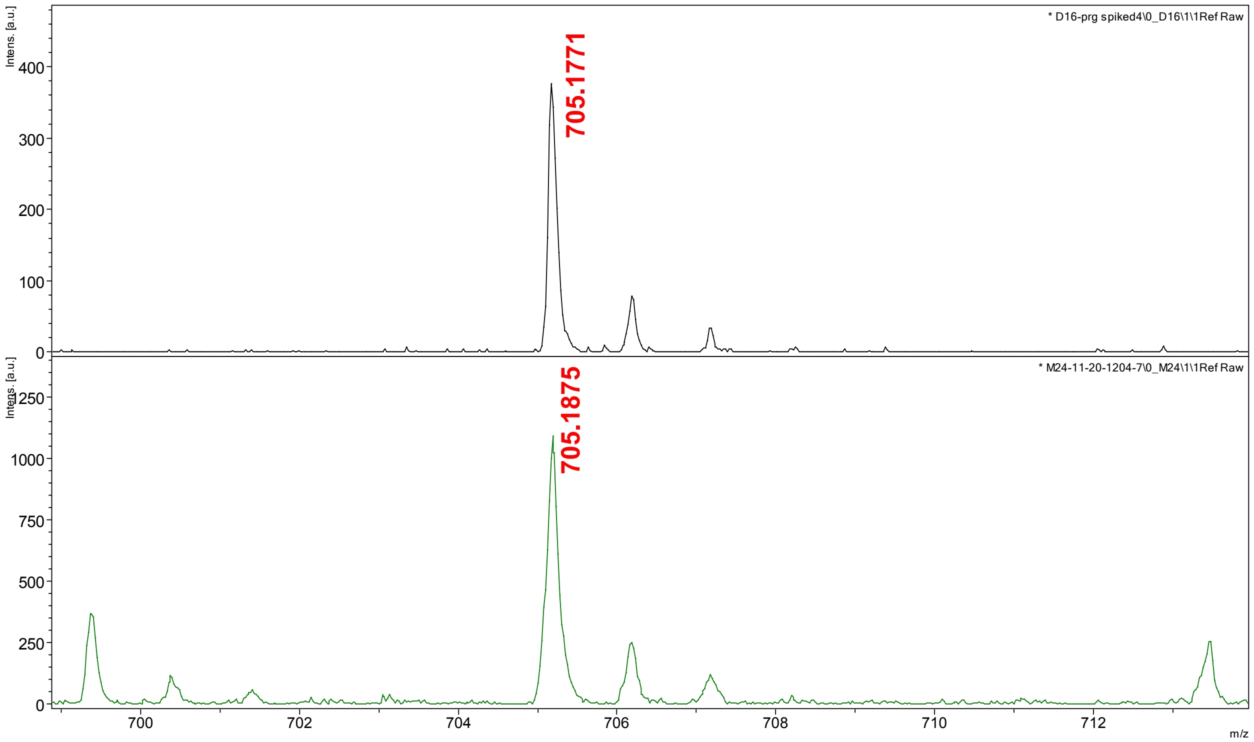

Figure S7 MALDI-MS spectra of tetrasaccharide+K in *Allium cepa* cytoplasm before (green) and after (black) spiking with dextran standard.

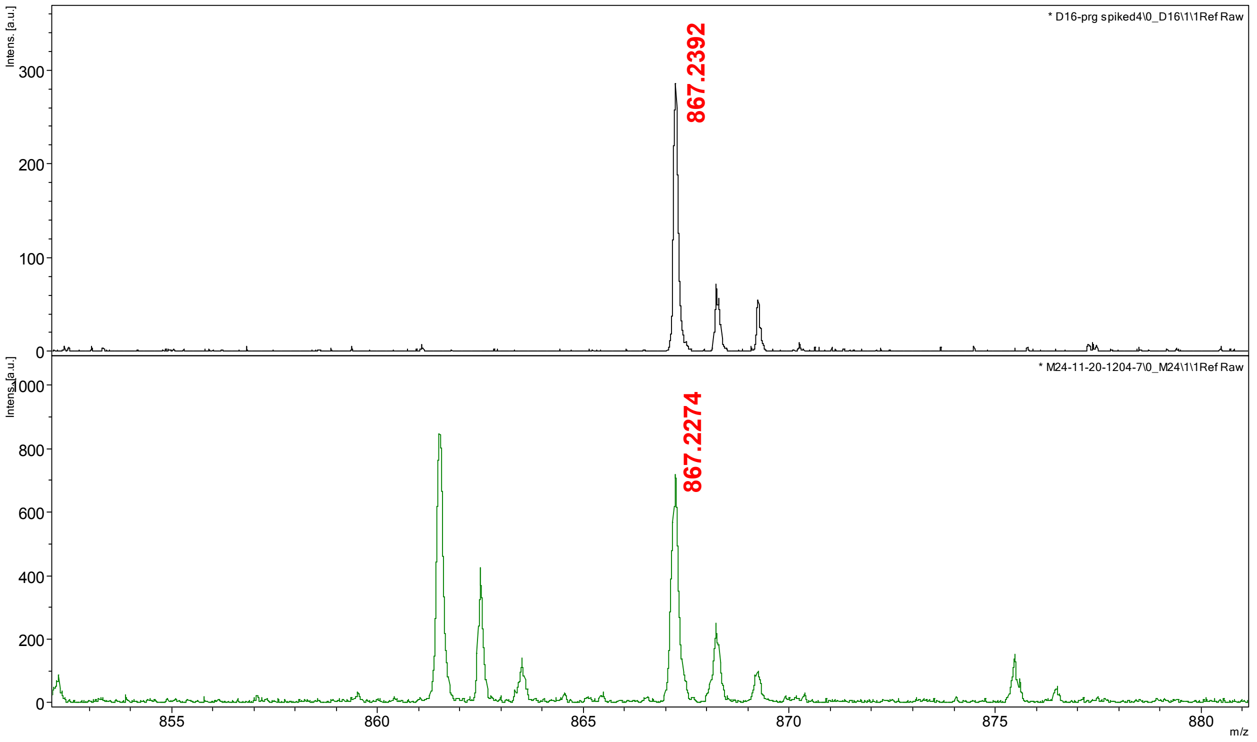

Figure S8 MALDI-MS spectra of pentasaccharide+K in *Allium cepa* cytoplasm before (green) and after (black) spiking with dextran standard.

Table S4 Tentative Peak assignments for lipids from *D. melanogaster* hemolymph

Assignment	Exact mass	Observed mass	Δppm
PS 32:4 [M+Na]	750.4317	750.4174	19.0
PS 32:3 [M+Na]	752.4473	752.4616	19.0
PS 32:2 [M+Na]	754.4630	754.4815	24.5
PC 34:3 [M+H]	756.5540	756.5410	17.2
PC 34:2 [M+H]	758.5694	758.5789	12.5
PC 34:1 [M+H]	760.6010	760.5923	11.4
PS 34:3 [M+Na]	780.4786	780.4902	14.9

PS 34:2 [M+Na]	782.4943	782.5068	16.0
PS 34:5 [M+K]	792.4212	792.4290	9.8
PS 34:4 [M+K]	794.4369	794.4491	15.3

Table S5 Tentative Peak assignments for lipids from Rat brain tissue section

Assignment	Exact mass	Observed mass	Δppm
PC 32:0 [M+H]	734.5694	734.5625	9.3
PC 34:1 [M+H]	760.5851	760.5852	0.1
PC 36:4 [M+H]	782.5694	782.5857	20.8
PC 36:1 [M+H]	788.6164	788.6247	10.5
